# Molecular detection of *Taenia* spp. in dogs’ feces in Zanjan Province, Northwest of Iran

**DOI:** 10.14202/vetworld.2017.445-449

**Published:** 2017-04-23

**Authors:** Mohammad Hasan Kohansal, Abbasali Nourian, Ali Haniloo, Asghar Fazaeli

**Affiliations:** Department of Parasitology and Mycology, School of Medicine, Zanjan University of Medical Sciences, Zanjan, Iran

**Keywords:** dog, *Echinococcus* spp, eggs, multiplex polymerase chain reaction, *Taenia* spp

## Abstract

**Aim:**

*Echinococcus* and *Taenia* spp. are important but neglected zoonotic helminths of dogs. Dogs as the most relevant definitive hosts harbor several species of *Taenia* and *Echinococcus* simultaneously in their gastrointestinal lumen which are morphologically indistinguishable. In this study, we used a multiplex polymerase chain reaction (PCR) method to identify Taeniid infections which seem to be highly distributed in the study region.

**Materials and Methods:**

A total of 450 dog fecal samples were collected from eight different areas of Zanjan province, northwest of Iran, and examined using a flotation method followed by multiplex PCR for detection and identification of parasites’ eggs.

**Results:**

Gastrointestinal parasites were found in 86 out of 450 fecal samples (19.1%) by microscopic examination. Taeniid eggs were observed in 5.6% of samples, containing 0.45%, 3.8%, and 1.3% *Echinococcus granulosus*, *Taenia* spp., and mix infection of both *E. granulosus* and *Taenia* spp., respectively. *Echinococcus multilocularis* was absent in the samples.

**Conclusion:**

A relatively low rate of *E*. *granulosus* (1.8%) was observed in this study. However, risks of this parasite should not be overlooked, and control programs need to be extended for this species and other Taeniid spp. In particular, dogs are recommended to be dewormed more frequently.

## Introduction

Dogs as animals that are involved with human life and environment harbor a number of important zoonotic helminth infections some of which are Taeniid cestodes [1,2]. The important species of Taeniidae family presented in dogs include *Echinococcus granulosus*, *Echinococcus multilocularis*, *Taenia ovis*, *Taenia multiceps*, and *Taenia hydatigena*. Among these parasites, two major species, *E*. *granulosus* and *E. multilocularis*, are highly important from a medical and public health standpoint [3-5]. There are different ways for parasite transmission; however, the ingestion of infective eggs is the major transmission route for intermediate and aberrant hosts. After accidental ingestion of *Echinococcus* eggs, it passes through canids feces and is transmitted to the intermediate hosts which finally develop echinococcosis cysts in organs, tissues, or body cavities [6,7].

Canids can pass several species of *Taenia* and *Echinococcus* eggs simultaneously. However, they are microscopically indistinguishable, and other methods are required for simple and reliable identification of the parasite species for effective diagnosis and treatment, as well as epidemiological survey and control programs [8]. Detection and differentiation of eggs from individual definitive hosts or feces collected in the field are essential challenge because they are sanitary indicators of the exposure level [9]. Despite post-mortem (necropsy) detection is highly sensitive and specific, it is evidently laborious, raises ethical issues, and is risky [10,11]. Two alternative approaches based on the detection of the parasitic copro-DNA molecules by polymerase chain reaction (PCR) and copro-antigens by enzyme-linked immunosorbent assay in animal fecal samples have been successfully developed and evaluated [12,13]. The detection and identification of copro-DNA may be advantageous over the detection of copro-antigens, as it provides the possibility of post-detection analysis of the sample DNA for precise identification of the proposed species. A number of techniques such as restriction fragment length polymorphism, direct comparison of PCR-amplified DNA sequences, random amplification of polymorphic DNA, single-strand conformation polymorphism, and microsatellite analysis have been employed for the identification of *Echinococcus* and *Taenia* spp. [8]. Studies showed that among different PCR methods, multiplex PCR targeting mitochondrial DNA is considered useful for the detection of Taeniid eggs [14]. Availability of the mitochondrial genome information for all *Echinococcus* species and several *Taenia* species provide a very rich resource of genetic information for molecular identification and discrimination [8]. Previous multiplex PCR studies targeting the mitochondrial DNA successfully amplified the DNA of all cysts and eggs and differentiated between *Echinococcus* spp., and *Taenia* spp. [10,15,16]. In the study area, northwest of Iran, there was no information regarding the infection rate of *Taeniid* spp. eggs in dogs’ feces dispersed in the environment to evaluate the potential risk of infection in human population living in the area.

The aim of this study was to detect the Taeniid eggs in dispersed feces of stray dogs and to identify different species, including zoonotic *E. granulosus* and *E. multilocularis*, and *Taenia* spp., in Zanjan province, northwest of Iran.

## Materials and Methods

### Ethical approval

The study proposal was reviewed and approved by the Institutional Research Ethics Committee at Zanjan University of Medical Sciences.

### Study area

**Figure-1:**
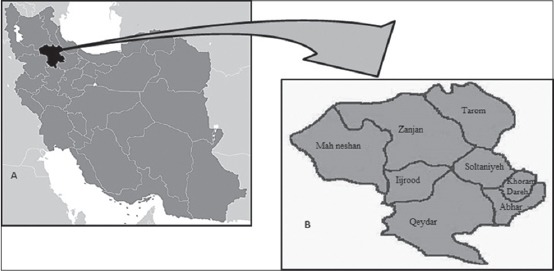
Map of Zanjan province in Iran and its regions, where dog fecal samples were collected.

Zanjan province is situated in the northwest of Iran between latitudes of 36°40′24″ and longitudes of 48°28′43″. It is divided into eight geographical realms with total area of 22164 km^2^ occupying 1.34% of Iran territory (Figure-1). Zanjan is one of the coldest provinces of Iran with an average minimum temperature of −19°C that drops to −27°C during the icy days. The average maximum temperature of Zanjan is around 27°C and the temperature rises to 32°C on hot days. The province is one of the important centers of agriculture and animal husbandry in the west of Iran. The number of livestock in the province is 2.366.411 animal [17].

### Collection and examination of dog fecal samples

The study was conducted between June and November 2015. A total of 450 samples of dog fresh feces were collected from streets in urban locations as well as farms in rural areas of different parts of the province. The fecal samples were placed in labeled “ziploc” bags and transported to the Research Laboratory at the Department of Parasitology, Faculty of Medicine, Zanjan University of Medical Sciences, on the day of collection and underwent microscopic examinations. All samples were examined with the naked eye for possible observation of adult helminths and proglottids of cestodes, followed by microscopic examination using formalin-ethyl acetate sedimentation concentration method. Consequently, to isolate Taeniid eggs, samples were frozen at −80°C for at least 3 days for safety reasons and then zinc chloride solution (specific density 1.45 g/ml) method was used as previously described [18]. Floated samples were passed through gauze and were centrifuged. The sediments were stored at −20°C until use.

### DNA extraction and multiplex PCR

The DNA of positive samples with *Taenia* spp. eggs was extracted using the QIAamp fast DNA stool Mini kit (Qiagen, Germany) according to the manufacturer’s protocol with slight modifications. First, 600 µl digestion buffer (100 mM NaCL, 10 mM Tris-HCL pH 8.0, and 25 mM EDTA) was mixed with 200 µl of each sediment obtained from flotation method. Glass pearls (0.45-0.52 mm diameter) were added to the mixtures, and the samples were vortexed for 10 min. Consequently, they were subjected to seven cycles of freeze/thaw using liquid nitrogen and boiling water for disrupting the egg walls. At this stage, 20 µl proteinase K (final concentration of 200 µg/mL) and 40 µl of 2% sodium dodecyl sulfate were added to each sample and placed in 60°C water bath for 16 h. Finally, the parasite DNA was extracted according to the manufacturer’s instructions. Concentration of the extracted DNAs was measured by Nanodrop (Thermo scientific 2000C). The extracted DNAs were stored at −20°C until analysis. Primers (targeting mitochondrial DNA), time and thermal conditions, and other parameters for multiplex PCR were used according to Trachsel *et al*. [14]. The primers used for multiplex PCR in this study are shown in Table-1. The amplification reactions were provided in 50 µl volume, containing 25 µl Master Mix (Ampliqon, Vietnam), 5 µl primers (2 µM of each primer, Cest1, Cest2, Cest3, Cest4, and 16 µM of primer Cest5 in H2O), 18 µl H_2_O, and 2 µl templates DNA. PCR cycling conditions consisted of one cycle of 15 min primary denaturation at 95°C; followed by 40 cycles of denaturing at 94°C for 30 s, annealing at 58°C for 90 s, and extension at 72°C for 10 s, ended with one cycle of final extension at 72°C for 7 min. Finally, 5 µl of each PCR product were analyzed by electrophoresis on 2% agarose gel stained with Safe Stain (SinaClon, Iran). A 100 bp ladder as molecular size marker was run together with samples to determine the fragment lengths. PCR products were visualized under ultraviolet light (UVIdoc, England).

**Table-1:** Primers, targets, and sequences applied for multiplex PCR.

Target species	Target gene	Primer designation	Sequences (5’-3’)	Amplicon size (bp)
*Echinococcus multilocularis*	nad1	Cest1	TGCTGATTTGTTAAAGTTAGTGATC	395
		Cest2	CATAAATCAATGGAAACAACAACAAG	
*Echinococcus granulosus*	rrnS	Cest4	GTTTTTGTGTGTTACATTAATAAGGGTG	117
		Cest5	GCGGTGTGTACMTGAGCTAAAC	
*Taenia* sp.	rrnS	Cest3	YGAYTCTTTTTAGGGGAAGGTGTG	267
		Cest5	GCGGTGTGTACMTGAGCTAAAC	

PCR=Polymerase chain reaction

## Results

**Figure-2:**
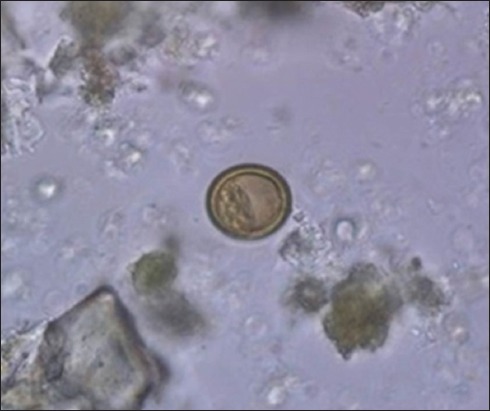
*Taenia/Echinococcus* eggs detected in dog fecal samples.

**Figure-3:**
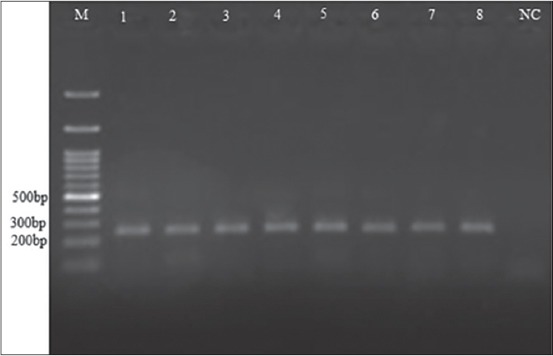
Gel electrophoresis of the polymerase chain reaction (PCR) products of Taenia spp. detected in dog feces. M is the 100 bp ladder. Lanes 1 to 8 are 267 bp PCR products representative of Taenia spp. NC is negative control.

**Figure-4:**
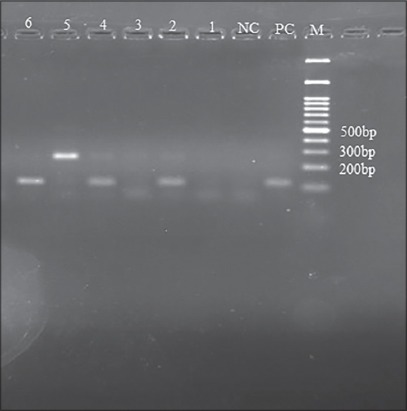
Gel electrophoresis of the polymerase chain reaction (PCR) products of mix infections with *Taenia* spp., and *Echinococcus granulosus* in dog feces. M is the 100 bp ladder. PC is positive control for *E. granulosus* (G1). NC is negative control. Lanes 1 to 6 are 267 bp and 117 PCR products of the samples.

A total of 86 out of 450 examined fecal samples (19.1%) contained at least one gastrointestinal parasite. Taeniid eggs were observed in 25 samples (Figure-2). They were subjected to multiplex PCR analysis. Amplification of a 117 bp fragment of rrnS target was observed in 0.45% (n=2) of the fecal samples, indicative of infection with *E. granulosus* eggs. A 267 bp fragment, indicative of *Taenia* spp. infection, was amplified in 3.8% (n=17) of the samples (Figure-3). There was no amplification of the 395 bp fragment of nad1 target in the 25 microscopically positive fecal samples, confirming no infection with *E. multilocularis*. In addition, 6 dog samples (1.3%) were coinfected with *E. granulosus* and other *Taenia* spp. (Figure-4).

## Discussion

The results of this study showed a relatively high rate of parasitic infection in dog feces dispersed in the environment of the study region in northwest of Iran, consisting of several parasite species, including *Echinococcus* and *Taenia* spp. Gastrointestinal parasites of dogs are of great importance because of the potential risk of transmission of zoonotic species to human in both rural and urban areas. Transmission of dog-related zoonotic parasites to humans primarily takes place through contact with the feces of the infected animals [1]. Information about the prevalence of such parasitic infections in dog fecal sources can be taken to control measure to minimize the risk of their transmission to humans. In this study, the overall prevalence of parasites was 19.1% among the 450 fecal samples analyzed. This infection rate was greater than what reported in China (12%) [19] and Kyrgyzstan (18%) [20], but less than that of Argentina (37.86 %) [21] and Spain (28%) [22]. Among parasites detected by flotation method, Taeniid eggs were observed in 5.6 % samples which was less than the infection rate reported by Rahimi *et al*. [16] in Mazandaran, north of Iran. The cestode family of Taeniidae contains 2 genera, *Taenia* and *Echinococcus*, which are closely related to each other, and their zoonotic importance made them the subject of intensive epidemiological studies [23]. The accurate diagnosis of Taeniid eggs provides key information to control the diseases. Stool exam is not accurate enough for epidemiological purposes, because the *Taeniidae* spp. eggs are extremely similar. Likewise, immunological methods (for copro-antigens detection) and serum antibodies are not sufficiently specific to differentiate these pathogens at species level [24]. To overcome these limitations, various molecular approaches, such as high-sensitivity Multiplex PCR technique, have been developed [14,16]. The results of multiplex PCR in our study showed a 0.45% infection rate with *E. granulosus*, 3.8% with *Taenia* spp., and 1.3% coinfection of *E. granulosus* and *Taenia*. In total, 1.8% of the examined samples were infected with important zoonotic species of *E. granulosus* in Zanjan province. In the studies performed in other areas of Iran, different infection rates of these species were reported using multiplex PCR technique. Mobedi *et al*. [25] and Beiramvand *et al*. [26] reported the rate of these infections as 23.7% and 26.3%, respectively. However, in another study conducted by Gholami *et al*. [27], no positive sample was found in stray dogs in northern part of Iran. There are several environmental and socioecological factors that are involved in the circulation of *E. granulosus* between hosts and can affect the prevalence of the infection. These include extensive sheep farming, home slaughter and dog feeding with animal viscera, dogs scavenging on infected carcasses, and improper disposal of carcasses [28]. Echinococcosis is endemic in Iran, and there are many reports from various parts of the country [29]. The prevalence rate of cystic echinococcosis has previously been reported in human population [30]. It was estimated that approximately 1% of admissions to surgical wards were related to cystic echinococcosis [31,32]. *E. multilocularis* is another parasite that can be found in dogs. In humans, the larval stage of *E. multilocularis* causes alveolar echinococcosis (AE), a space-occupying lesion, which is lethal if untreated. AE, as a sporadic human disease, has been distributed over the most of the northern hemisphere and some countries of the Middle East [7]. In Iran, a few studies were conducted on *E. multilocularis* infection [26,33]; however, no study with this concern has been performed in Zanjan province. In this study, no 395 bp amplicon, representative of *E. multilocularis* eggs, was observed in the Taeniid-positive fecal samples of dogs. Our result is in line with a couple of studies performed in north of Iran, in which no *E. multilocularis* was detected in dog fecal samples [16,25]. Studies have shown that median estimate of the total numbers of AE cases in the world is 18,235 per year [34]. It should be noted that this species usually has a sylvatic life cycle, involving foxes rather than dogs as the definitive host [6,35]. In spite of no detection of *E. multilocularis* in this study, it cannot be claimed that this infection is completely absent in the area, and larger scale studies focusing on other hosts (definitive and intermediate) are needed. Al-Sabi *et al*. [10] developed a multiplex-PCR to differentiate *Taenia* species in rodents and carnivores. Compared to morphological methods, their assay had a significantly higher sensitivity as they identified 31 of 32 *Taenia* metacestodes from rodents, whereas only 14 samples were specifically identified by morphological methods. Yamasaki *et al*. [15] devised a multiplex PCR for differential diagnosis of taeniasis and cysticercosis of humans. They successfully amplified 827 and 269 bp products for *T. saginata* and Asian *Taenia*, respectively, using mixed species- or genotype-specific primer sets.

## Conclusion

It was confirmed that the Taeniid infections are endemic in dogs in Zanjan province, northwest of Iran. Dog feces dispersed in the environment, particularly those of stray dogs, are an important source of transmissible infection to ruminants as intermediate hosts and human as an accidental host. Among these, *E. granulosus* eggs, which are the cause of a considerable number of human cystic hydatidosis, are of greater importance. Differences between infection rates of this parasite in different parts of Iran depend on many factors such as definitive and intermediate hosts. Therefore, more studies are required to determine precise prevalence of these parasites in all hosts in different areas around the study regions.

## Authors’ Contributions

MHK provided the research proposal, collected the samples and performed laboratory works. AN and AF supervised the project. AH was a scientific and lab diagnostic advisor to the project. The manuscript was written by MHK, finally revised by AF. All authors read and approved the final manuscript.
